# The European Society of Human Reproduction and Embryology guideline for the diagnosis and treatment of endometriosis: an electronic guideline implementability appraisal

**DOI:** 10.1186/1748-5908-6-7

**Published:** 2011-01-19

**Authors:** Lotte JEW van Dijk, Willianne LDM Nelen, Thomas M D'Hooghe, Gerard AJ Dunselman, Rosella PMG Hermens, Christina Bergh, Karl G Nygren, Arnold HM Simons, Petra de Sutter, Catherine Marshall, Jako S Burgers, Jan AM Kremer

**Affiliations:** 1 Department of Obstetrics & Gynaecology, Radboud University Nijmegen Medical Centre, Nijmegen, The Netherlands; 2 Department of Obstetrics & Gynaecology, University Fertility Center, Gasthuisberg University Hospital, Leuven, Belgium; 3 Department of Obstetrics & Gynaecology, Maastricht University Medical Centre and Research Institute GROW, Maastricht University, Maastricht, The Netherlands; 4 Scientific Institute for Quality of Healthcare and Dutch Institute for Healthcare Improvement CBO, Radboud University Nijmegen Medical Centre, Nijmegen, The Netherlands; 5 Department of Obstetrics & Gynaecology, Institution of Clinical Sciences, Sahlgrenska Academy, Göteborg University, Göteberg, Sweden; 6 IVF Clinic, Queen Sophia Hospital, Stockholm, Sweden; 7 Department of Obstetrics & Gynaecology, University Medical Centre, Groningen, The Netherlands; 8 Department of Obstetrics & Gynaecology, Infertility Centre, Gent University Hospital, Gent, Belgium; 9 Independent Guideline Adviser, New Zealand

## Abstract

**Background:**

Clinical guidelines are intended to improve healthcare. However, even if guidelines are excellent, their implementation is not assured. In subfertility care, the European Society of Human Reproduction and Embryology (ESHRE) guidelines have been inventoried, and their methodological quality has been assessed. To improve the impact of the ESHRE guidelines and to improve European subfertility care, it is important to optimise the implementability of guidelines. We therefore investigated the implementation barriers of the ESHRE guideline with the best methodological quality and evaluated the used instrument for usability and feasibility.

**Methods:**

We reviewed the ESHRE guideline for the diagnosis and treatment of endometriosis to assess its implementability. We used an electronic version of the guideline implementability appraisal (eGLIA) instrument. This eGLIA tool consists of 31 questions grouped into 10 dimensions. Seven items address the guideline as a whole, and 24 items assess the individual recommendations in the guideline. The eGLIA instrument identifies factors that influence the implementability of the guideline recommendations. These factors can be divided into facilitators that promote implementation and barriers that oppose implementation. A panel of 10 experts from three European countries appraised all 36 recommendations of the guideline. They discussed discrepancies in a teleconference and completed a questionnaire to evaluate the ease of use and overall utility of the eGLIA instrument.

**Results:**

Two of the 36 guideline recommendations were straightforward to implement. Five recommendations were considered simply statements because they contained no actions. The remaining 29 recommendations were implementable with some adjustments. We found facilitators of the guideline implementability in the quality of decidability, presentation and formatting, apparent validity, and novelty or innovation of the recommendations. Vaguely defined actions, lack of facilities, immeasurable outcomes, and inflexibility within the recommendations formed barriers to implementation. The eGLIA instrument was generally useful and easy to use. However, assessment with the eGLIA instrument is very time-consuming.

**Conclusions:**

The ESHRE guideline for the diagnosis and treatment of endometriosis could be improved to facilitate its implementation in daily practice. The eGLIA instrument is a helpful tool for identifying obstacles to implementation of a guideline. However, we recommend a concise version of this instrument.

## Background

Clinical guidelines are important tools for improving the quality, effectiveness, and appropriateness of healthcare [1-5]. They are intended to bridge the gap between research and practice and to assist clinicians and patients in clinical decision making [2,6,7]. Moreover, they can reduce the use of unnecessary diagnostic tests and treatments [8]. However, adherence to guidelines is often poor and not self-evident [9-13]. Implementation of guidelines requires 'turning changes in attitude and knowledge into changes in medical practice' [14]. To improve guideline adherence and consequently healthcare, the implementability of the guidelines should be taken into account [15,16].

Factors that influence the implementability of guidelines can be divided into facilitators that promote implementation and barriers that oppose implementation [17]. Various studies describe these factors [17-20], which can be classified as factors relating to physicians or patients, to the methodological quality of a guideline (including the clarity and applicability of its recommendations), and to the external context (*e.g.*, legislation and required facilities) [21].

Implementation of guidelines is significant in many medical disciplines and is especially important in subfertility care because it is concerned with social, financial, legal, and ethical implications [22]. Subfertility is defined as lack of conception after at least one year of unprotected intercourse [23]. Approximately 80 million people worldwide suffer from this disorder [24]. More than half of subfertile couples seek medical care [25]. Clinical guidelines can be helpful to set standards and to organise the care properly. Emslie and coworkers showed improvements in the process of subfertility care with the use of guidelines [1]. Collaboration in developing the European subfertility guidelines could improve their scientific validity and promote international consensus on their clinical content [4,26,27]. This may help reduce practice variation and quality defects at an international level.

The European Society for Human Reproduction and Embryology (ESHRE) is one of the international organisations that participates in the process of developing international clinical practice guidelines in the area of reproductive medicine [28]. Nelen and coworkers evaluated 11 ESHRE guidelines with the validated Appraisal of Guidelines for Research and Evaluation (AGREE) instrument [29,30]. The methodological quality of most of these clinical ESHRE guidelines was poor, while the quality of five of the guidelines was better. The ESHRE guideline for the diagnosis and treatment of endometriosis had the highest methodological quality. However, data about the implementability of these guidelines are not available. Such data are crucial for better application of the ESHRE guidelines.

Various methods have been developed for assessing guideline implementability [31,32]. Shiffman and coworkers recently developed an instrument, the guideline implementability appraisal (GLIA) instrument, for which evidence of content validity and support for construct validity were obtained [33]. The instrument contains a series of validated questions for assessing the relative ease of implementation of guideline recommendations. It identifies potential obstacles to implementation that are primarily intrinsic to the guideline. This makes the instrument useful for guideline developers to remedy defects in guidelines and for guideline implementers to identify barriers [33]. Moreover, an electronic version of this tool has been developed: the eGLIA instrument.

We investigated the implementability of the ESHRE guideline on endometriosis with the eGLIA instrument to identify potential barriers to implementation and to refine the guideline. We also evaluated the eGLIA instrument for its usefulness and feasibility as an appraisal tool for improving the implementability of a guideline.

## Methods

### Clinical practice guideline

We reviewed the ESHRE guideline for the diagnosis and treatment of endometriosis, which contains 36 recommendations (Additional File 1, Appendix 1). We used the published paper version of the guideline for our appraisal [34].

### Appraisal instrument

The GLIA instrument, developed by Shiffman and coworkers, was used to identify obstacles to implementation [33] (Additional file 2, Appendix 2). The first part of the GLIA instrument consists of seven global dimension questions (Q1-Q7) that relate to the guideline as a whole. The second part of the instrument consists of 24 questions for assessing the implementability of each individual recommendation (Q8-Q31). These questions are grouped into nine dimensions: decidability (*n *= 3), executability (*n *= 2), effect on process of care (*n *= 2), presentation and formatting (*n *= 2), measurable outcomes (*n *= 2), apparent validity (*n *= 2), novelty (*n *= 3), flexibility (*n *= 4), and computability (*n *= 4). The last four questions rating computability are optional and only apply when an electronic implementation is planned. All items have four response categories: 'Y' (yes), 'N' (no), 'NA' (not applicable), and '?' (unsure). The GLIA instrument provides additional space for comments about how a recommendation fulfils or fails to fulfil a criterion. Additional File 3, Appendix 3 gives an example of scoring. The GLIA instrument does not provide an overall judgement of the implementability of the guideline as a whole. Therefore, we added an extra question to seek the general opinion of the appraisers about the implementability of the guideline. We used a five-point Likert scale (1 = definitely not implementable, 5 = definitely implementable) to assess the rating.

We used eGLIA, the electronic version of the GLIA instrument http://nutmeg.med.yale.edu/eglia/. This electronic tool has an advantage over the paper version because it is useful and feasible to use with limited training and time [35]. Moreover, the electronic version offers automatic data storage, which was especially advantageous for our appraisers coming from different countries.

### Composition of the panel of appraisers

We composed a balanced panel of 10 clinical and methodology experts. We selected six clinical experts: two developers of the guideline about endometriosis (TD and GD), one expert on endometriosis (AS), and three experts in subfertility care from the Special Interest Group on Safety and Quality in Assisted Reproductive Technology (SIG SQUART) from ESHRE (CB, KN, and PS). Furthermore, two researchers from the department of Obstetrics and Gynaecology (LD and WN) and two experts in quality of care (JB and CM), one of whom had special expertise with the eGLIA instrument (CM), participated in this study. The appraisers came from Belgium, Sweden, the Netherlands, and New Zealand.

### Appraisal of the guideline

We asked the panel members to read the ESHRE guideline for the diagnosis and treatment of endometriosis and to assess it with the eGLIA instrument using their own computers.

We collected the individual scores of the participants and determined the discrepancies in scoring. We sent every appraiser an overview of his or her answers and the frequencies in the other participants' scores. This overview made differences in scoring clear for each assessor. There was a one-hour telephone conference to discuss the discrepancies between assessments and to come to a final score, as the eGLIA tool indicates to do. The content experts helped resolve questions answered with '?' (unsure) in this phone conference. Then the participants decided conclusively whether a recommendation had met a particular criterion or failed it. Each final decision was based on agreement reached by an absolute majority of the participants (difference ≥2).

### Analysis

Items voted for by an absolute majority of participants were marked. Items with the answer 'No' were seen as barriers. Items with the answer 'Yes' were seen as facilitators. Items with a slight majority (one-point difference) were treated as borderline barriers. Questions that were answered 'No' and did not satisfy the criterion were listed as barriers, and recommendations for adjustments or changes were made.

### Process evaluation of eGLIA

The appraisers individually completed a questionnaire (12 questions) about their experience with the eGLIA instrument immediately after they used it. The questionnaire included items about time investment, clarity and usability of the instrument, and relevance of the eGLIA tool questions. The questions were evaluated on a five-point Likert scale ranging from 1 (strongly disagree) to 5 (strongly agree). We performed descriptive statistical analyses with SPSS for Windows Release 14.0 Standard Version (SPSS Inc., Chicago, IL, USA).

## Results

### Appraisal of the guideline

Eight of the 10 participants appraised all 36 recommendations with 24 questions. One appraiser assessed 25 recommendations, and one participant appraised only 7 recommendations because of lack of time.

In the final report (Additional File 4, Appendix 4), 69 questions are marked as barriers (in red) and 501 as facilitators (in green). Twelve borderline barriers (doubtful items with only one-point difference) were marked separately (in orange) with inside in the table written the tendency toward which answer.

The guideline included five 'recommendations' (R14, R26, R31, R34, and R36) that did not have a described condition or action, the so-called nonrecommendations. These nonrecommendations were statements or observations that could not be appraised with the eGLIA instrument. Therefore, we excluded them from further analyses.

When we analysed the global dimension, we found three barriers to implementation (Q3-Q5). First, the guideline did not address strategies for implementation (Q3), although it seemed that dissemination of the guideline had been undertaken with an online version. Second, there was no tool for application (Q4) available, such as a summary document. The electronic version on the ESHRE website http://www.guidelines.endometriosis.org/ provided access to a concise summary and supporting documentation, but the paper version of the guideline did not refer to this. Third, the differences in the importance of the recommendations (Q5) were only described at the level of evidence. A clear presentation or formatting reflecting the differences was lacking.

Regarding the individual recommendations, two were straightforward to implement (R1 and R12). The remaining 29 recommendations contained one or more barriers.

### Facilitators

The guideline scored very well on four dimensions, which can be considered implementation facilitators. First, the dimension of decidability (Q8-Q10) had positive scores for almost all recommendations. The description of the conditions and their mutual relations were very clear. All recommendations were easily identifiable because they were summarised in frames. Only two recommendations (R19 and R32) had a vague definition of the stated condition. For instance, the phrase 'depending on the severity of the disease' would need further specification (R19).

Second, the recommendations were as concise as possible and their presentation and formatting (Q15 and Q16) provided good visibility.

Third, the apparent validity (Q19 and Q20) was scored as a facilitator due to the structured reporting of the evidence and its quality linked to the individual recommendations.

Fourth, in the dimension of novelty/innovation (Q21-Q23), almost all recommendations were feasible without the need of new skills or knowledge (Q21). Moreover, the guideline considered the existing attitudes and beliefs of the intended users of the guideline (Q22 and Q23). However, R35 appeared incompatible with existing attitudes and beliefs of the guideline's intended users because it favoured complementary medicine.

### Barriers

Four barriers to implementation were identified. First, the appraisers found that executability (Q11 and Q12) was a barrier in various recommendations (R7, R9, R10, R13, R15, and R33) because they were vague in their descriptions of the recommended actions. Formulations such as 'consideration should be given' did not make clear whether the action should be carried out or not. In addition, information about how a certain action should be performed was missing. Measuring adherence to such recommendations is difficult.

Second, the effect on the process of care (Q13 and Q14) was identified as a barrier. Four recommendations (R9, R11, R21, and R30) included actions that needed extra equipment, staff, or provider time to make them implementable. For example, not all hospitals have magnetic resonance imaging or facilities for in vitro fertilisation available.

Third, the lack of clear measures (Q17 and Q18) was a barrier in seven recommendations (R5, R8, R10, R19, R23, R24, and R35). There were no criteria for measuring adherence to these recommendations, which could complicate the monitoring of endometriosis care.

Fourth, the flexibility (Q24-Q27) was found to be a barrier. Some recommendations (R7, R8, R10, R11, R15-R17, R19, R33, and R35) lacked specific patient or practice characteristics to enable individualisation of care (Q24). Most recommendations (R2-R10, R13, R15-R25, R27-R30, R32, R33, and R35) did not consider coincident drug therapy and common comorbid conditions (Q25). Furthermore, the incorporation of patient preference (Q27) formed a barrier. R33 and R35 considered this preference but did not propose any mechanisms to implement the preference in practice. An exception to flexibility as a barrier was the strength of the recommendations (Q26), which the guideline developers stated explicitly with the classification of the recommendations.

We excluded the four optional items from the dimension computability (Q28-Q31) from further analysis because no electronic implementation was planned. At the time of our study, information technology support systems were not available to implement the guideline.

Of the 36 recommendations, 15 were graded with evidence strength A. These recommendations (R6-R8, R16-R18, R20, R22, R24, R25, R27-R29, R31, and R33) had significantly fewer individual barriers for implementation than the remaining recommendations did (Table 1). Recommendations graded A had 26 barriers in 260 items (10%) versus 52 barriers in 315 items (16.5%) at levels B, C, D and the good practice points (*p *= .02; odds ratio = 0.5 [95% confidence interval, 0.3-0.9]).

**Table 1:** Barriers related to the grade strength of the evidence

Grading	Items	Barriers	Proportion of barriers to items (in percentage)	Total (in percentage)
A	260	26	10.0	26/260 (10.0)

B C D GPP	19 51 19 226	3 5 4 40	15.8 9.8 21.1 17.7	52/315 (16.5)

Strength of evidence

Grade A: Directly based on level 1 evidence

Grade B: Directly based on level 2 evidence or extrapolated recommendation from level 1 evidence

Grade C: Directly based on level 3 evidence or extrapolated recommendation from either level 1 or level 2 evidence

Grade D: Directly based on level 4 evidence or extrapolated recommendation from either level 1, 2, or 3 evidence

Grade GPP: Good practice point based upon the views of the Guideline Development Group

Hierarchy of evidence

Level Evidence

1a Systematic review and meta-analysis of randomised controlled trials

1b At least one randomised controlled trial

2a At least one well-designed controlled study without randomisation

2b At least one other type of well-designed quasi-experimental study

3 Well-designed, nonexperimental, descriptive studies, such as comparative studies, correlation studies, or case studies

4 Expert committee reports or opinions and/or clinical experience of respected authorities

### General implementability of the guideline

The median score for the additional question assessing the implementability of the guideline was 4, ranging from 2 (probably not implementable) to 5 (definitely implementable). Six appraisers (60%) thought that the guideline was probably implementable (with some adjustments) or definitely implementable. One participant considered the guideline as probably not implementable.

### Process evaluation of eGLIA

On average, the time the participants spent completing the appraisal (response 8 of 10) was four hours (range: three to eight hours). The average time needed to complete one recommendation was 10 minutes (range: 5 to 24 minutes). The answering became easier and quicker as more recommendations were appraised.

Most participants (60%) found the explanation of the GLIA dimensions and the use of the eGLIA tool clear (Table 2). However, they commented that more scoring examples would have been helpful. The general opinion was that the eGLIA tool was easy to use (70%) and functional for its purpose. Most questions were appraised with an agreement of more than 60% (for the answers 'agree' or 'strongly agree'). Identifying obstacles to implementation and judging the recommendations systematically were consistently appraised with close agreement (80% and 90%, respectively). There was wide variation in the understanding and application of the tool questions. Appraisers reported that several questions in the eGLIA instrument were not very clear or that they had to read them several times to understand the meaning. In addition, the participants stated that appraising a large number of questions was boring and too time consuming.

**Table 2:** Process evaluation of eGLIA

Questions (*n *= 10)	Answers		
	**D**	**N**	**A**

The explanation of the GLIA dimensions is clear	2	2	6
The explanation of the use of the eGLIA tool is clear	0	1	9
The eGLIA tool is easy to use	2	1	7
The tool questions are easy to understand and apply	2	6	2
The tool questions were relevant to assessing implementability	0	4	6
The eGLIA tool helped identify obstacles to implementation	1	1	8
The eGLIA helped to judge recommendations systematically	0	1	9
Will use the eGLIA more often	1	3	6

eGLIA = electronic guideline implementability appraisal; D = disagree or strongly disagree; N = neutral; A = agree or strongly agree.

## Discussion

The aim of this study was to investigate the implementability of the ESHRE guideline for the diagnosis and treatment of endometriosis with the aid of the eGLIA tool. In general, the appraisers considered the guideline implementable in daily practice. However, they identified important barriers to implementation for some recommendations. This shows that barriers to implementation exist even in guidelines that are rated as high-quality guidelines. Nonetheless, implementability must be differentiated from guideline quality. Quality is generally assessed for the guideline as a whole and determines the scientific validity of guidelines. Implementability is one component of guideline quality, and its assessment is applied to individual recommendations within a guideline.

Implementation of the guideline would be improved if a description of the implementation strategies was included. The addition of an application tool for physicians as well as for patients, *e.g.*, a summary document and a 'coping with endometriosis' leaflet, would also likely enhance implementation. Furthermore, we advise clearly displaying the most important recommendations as key recommendations at the end of the guideline.

Appraisal of the implementability of individual recommendations revealed important barriers that could be useful in designing implementation strategies and in updating the guideline. Recommendations could be reformulated to optimise their use in daily practice. Using a standard format or template for formulating recommendations could improve their implementability. The ESHRE has produced a manual on guideline development http://www.eshre.eu/ESHRE/English/Specialty-Groups/SIG/Safety-Quality-in-ART/Manual-for-ESHRE-Guideline-Development/page.aspx/254 comparable to the manuals of the National Institute for Health and Clinical Excellence [36] and the American Heart Association [37]. The manual states that recommendations should be stand-alone texts (*i.e.*, independent from headings), and they should be as concise but as detailed as possible. Each recommendation should be a description about who does what for whom, when, and how. Standard phrases are suggested to overcome misunderstandings and confusion. A guiding structure of developing guidelines and writing recommendations will help prevent vaguely formulated recommendations and 'nonrecommendations'. Ideally, a nonrecommendation should be restated as a recommendation with conditions and actions if possible. If this is not possible, the information in the statement of the nonrecommendation can be added in the supporting text. This way, the information is retained but not listed as a recommendation. Furthermore, conditions and actions should be defined concretely so that only one interpretation is possible. For example, in R33, 'prolonged treatment' does not specify what duration of treatment could be classified as 'prolonged'. As seen from the results, grade A recommendations have fewer barriers than those with a lower grade. This is most likely because a grade A recommendation has a clearer evidence base and can therefore be written unambiguously. A recommendation should include clearly defined, measurable outcomes. For example, in R24, 'the effectiveness of hormonal treatment' could be stated more explicitly, *e.g.*, 'its effectiveness on achieving pregnancy' or 'its effectiveness on giving birth'.

The implementability of a guideline should be considered in all phases of its development, including the scoping phase; the evidence review; and the dissemination, adoption, and use of the guideline in practice [15,38]. Applying guidelines requires good preparation, with a detailed analysis of the target group, patient involvement, systematic approach, and structured phrasing of the recommendations [7,12,39].

### Process evaluation of eGLIA

The second aim of this study was to evaluate the usefulness and feasibility of the eGLIA instrument. The results of this study indicate that the tool is useful in identifying barriers to implementation and in appraising the individual recommendations systematically. This is in line with Hill and coworkers' study [40]. Moreover, the web-based eGLIA appraisal facilitates international collaboration and the availability of international guidelines. The appraisers were widely distributed geographically. However, the eGLIA tool made it easy to collect and analyse the scores and to create a final report. This report is helpful for the adjustment of certain recommendations in a guideline to improve their implementability without the need for developing a new guideline. This obviates duplication of effort [41]. The eGLIA instrument is a tool that should be applied to each individual recommendation. It gives good insight into the barriers for implementation per recommendation. The eGLIA instrument is not intended for assessment of the implementability of the whole guideline, however, such an assessment would be an interesting addition. A ranking of the implementability of the individual recommendations could be considered, but this alone would not be accurate because some recommendations are substantially more important than others.

A limitation of the eGLIA tool is the time necessary for assessment. The eGLIA tool is probably unsuitable for guidelines with many recommendations. This leads to the question of practical use, in other words, the implementability of the eGLIA instrument itself for guidelines with many recommendations. To reduce the appraisal time, we suggest the development of a concise version of the current eGLIA instrument. For example, some of the tool's specific questions could be stated as general questions in the global dimension of the guideline as a whole (Q15, Q16, Q19, Q20, and Q23) because these questions -about format, validity, and patient expectations- often have equal scores for all recommendations. Other questions could be removed, as they have limited additional value. For example, Q27 is about patient preference, which is always considered and need not be asked generally. Another possibility is short questions with marking of keywords, which would reduce the reading time. Providing examples of 'good' recommendations and 'bad' recommendations would facilitate the scoring process.

### Limitations of the study

First, most participants did not have any experience with the eGLIA instrument. Therefore, they needed time to understand the items and to learn about assessing the recommendations. This is evident in the results: answering became easier and quicker as more recommendations were appraised. One participant (CM) had more experience with the eGLIA instrument. She could give directions and explain common problems in interpreting the questions. A training workshop might be helpful before starting a formal appraisal with the eGLIA instrument.

Second, the process evaluation of the eGLIA instrument was limited by the number of users (10) and the number of guidelines (one). Formal validation would need a larger group of appraisers and more guidelines in different health areas. For international validation, translations of the instrument and translation protocols should be developed. However, the study questionnaire has revealed an interesting view of the use and feasibility of the appraisal instrument.

Third, we investigated primarily factors intrinsic to the guideline. We did not consider external factors, such as organisational factors and environmental factors (*e.g.*, lack of time and lack of resources). A supplementary study could investigate these factors, possibly with a focus group or individual interviews of patients and professionals.

Fourth, both the appraisal of the guideline and the evaluation of the eGLIA tool were involved. Negative criticism of the eGLIA instrument may have interfered with the reliability of the guideline evaluation. However, the appraisers found the eGLIA tool useful and feasible for its purpose. We therefore consider the appraisal of the guideline valid.

The guideline developers and the eGLIA developers received feedback. The ESHRE guideline for the diagnosis and treatment of endometriosis will be revised in light of the results of this study.

## Conclusions

The ESHRE guideline for the diagnosis and treatment of endometriosis has some intrinsic barriers to implementation, which could be overcome by more accurate and systematic phrasing of the recommendations. For the future development of ESHRE guidelines and other guidelines, we recommend taking implementability issues into account at the time of the drafting of the guideline. The eGLIA tool might be useful and feasible for this purpose. However, we also advise development of a concise version of the eGLIA instrument.

## Competing interests

The authors declare that they have no competing interests.

## Authors' contributions

LD participated in the design of the study and elaborated it, appraised the guideline with eGLIA, participated in the telephone conference, did the statistical analysis, and drafted the manuscript. WN conceived of the study, participated in its design and coordination, appraised the guideline with eGLIA, and participated in the telephone conference and the revision the manuscript. TD, GD, CB, KN, AS, PS, and CM appraised the guideline with eGLIA and participated in the telephone conference and the revision of the manuscript. RH participated in the design of the study and the revision of the manuscript. JB participated in the design of the study, appraised the guideline with eGLIA, and participated in the telephone conference and the revision of the manuscript. JK coordinated the study, approached participants, presided over the telephone conference, and took part in the revision of the manuscript. All authors read and approved the final version of the manuscript.

## Supplementary Material

Additional file 1 Appendix 1 - Recommendations of the ESHRE guideline for the diagnosis and treatment of endometriosis. Click here for file

Additional file 2 Appendix 2 - Questions of the guideline implementability appraisal (GLIA) instrument. Click here for file

Additional file 3 Appendix 3 - Example of scoring. Click here for file

Additional file 4 Appendix 4 - Final report. Click here for file
