# Supplementary material for: The European Society of Human Reproduction and Embryology guideline for the diagnosis and treatment of endometriosis: an electronic guideline implementability appraisal
Source: Implement Sci. 2011 Jan 19;6:7. doi: 10.1186/1748-5908-6-7 (PMC3034686; doi:10.1186/1748-5908-6-7)
Supplement: Additional file 1 — Appendix 1 - Recommendations of the ESHRE guideline for the diagnosis and treatment of endometriosis. [file 1748-5908-6-7-S1.PDF]

Appendix 1

Recommendations of the ESHRE guideline for the diagnosis and treatment of endometriosis

		Strength of evidence
R1	Deeply infiltrating nodules are most reliably detected when clinical examination is performed during menstruation (Koninckx *et al.*, 1996).	C
R2	For a definitive diagnosis of endometriosis, visual inspection of the pelvis at laparoscopy is the 'gold standard' investigation, unless disease is visible in the vagina or elsewhere.	C
R3	Positive histology confirms the diagnosis of endometriosis; negative histology does not exclude it. Whether histology should be obtained if peritoneal disease alone is present is controversial: visual inspection is usually adequate but histological confirmation of at least one lesion is ideal. In cases of ovarian endometrioma (>3 cm in diameter), and in deeply infiltrating disease, histology **should be obtained** to identify endometriosis and to exclude rare instances of malignancy.	GPP
R4	If the patient wants pain symptoms suggestive of endometriosis to be treated without a definitive diagnosis, then a therapeutic trial of a hormonal drug to reduce menstrual flow is appropriate (see 'Empirical treatment' section).	GPP
R5	The management of severe/deeply infiltrating endometriosis is complex. Therefore, if disease of such severity is suspected or diagnosed, referral to a centre with the necessary expertise to offer all available treatments in a multi-disciplinary context, including advanced laparoscopic surgery and laparotomy, is strongly recommended.	GPP
R6	Compared to laparoscopy, transvaginal ultrasound (TVS) has no value in diagnosing peritoneal endometriosis, but it is a useful tool both to make and to exclude the diagnosis of an ovarian endometrioma (Moore *et al.*, 2002). TVS may have a role in the diagnosis of disease involving the bladder or rectum.	A
R7	Compared to laparoscopy, magnetic resonance imaging (MRI) has limited value as a diagnostic tool for endometriosis (Ang *et al.*, submitted).	A
R8	Serum CA-125 levels may be elevated in endometriosis. However, compared to laparoscopy, measuring serum CA-125 levels has no value as a diagnostic tool (Mol *et al.*, 1998).	A
R9	If there is clinical evidence of deeply infiltrating endometriosis, ureteral, bladder and bowel involvement should be assessed. Consideration should be given to performing MRI or ultrasound (transrectal and/or transvaginal and/or renal), with or without intravesical pressure (IVP) and barium enema studies depending upon the individual circumstances, to map the extent of disease present, which may be multi-focal.	GPP
R10	Local guidelines for the management of suspected ovarian malignancy should be followed in cases of ovarian endometrioma. Ultrasound scanning ± serum CA-125 testing is usually used to try to identify rare instances of ovarian cancer; however, CA-125 levels can be elevated in the presence of endometriomas.	GPP

R11	Good surgical practice is to document in detail the type, location and extent of all lesions and adhesions in the operative notes; ideal practice is to record the findings on video or DVD.	GPP
R12	There is insufficient evidence to justify timing the laparoscopy at a specific time in the menstrual cycle, but it should not be performed during or within 3 months of hormonal treatment so as to avoid under-diagnosis.	GPP
R13	All classification systems for endometriosis are subjective and correlate poorly with pain symptoms, but may be of value in infertility prognosis and management (Chapron *et al.*, 2003b; D'Hooghe *et al.*, 2003).	C
R14	At laparoscopy, deeply infiltrating endometriosis may have the appearance of minimal disease, resulting in an underestimation of disease severity (Koninckx *et al.*, 1994).	C
R15	Empirical treatment for pain symptoms presumed to be due to endometriosis without a definitive diagnosis includes counselling, adequate analgesia, nutritional therapy, progestagens or the combined oral contraceptive (COC). It is unclear whether the COC should be taken conventionally, continuously or in a tricycle regimen. A GnRH agonist may be taken but this class of drug is more expensive, and associated with more side-effects and concerns about bone density.	GPP
R16	Non-steroidal anti-inflammatory drugs (NSAID) may be effective in reducing endometriosis-associated pain (Kauppila *et al.*, 1979; Ylikorkala and Viinikka, 1983; Kauppila and Ronnberg, 1985).	A
R17	Suppression of ovarian function for 6 months reduces endometriosis-associated pain. The hormonal drugs investigated—COC, danazol, gestrinone, medroxyprogesterone acetate and GnRH agonists—are equally effective but their side-effects and cost profiles differ (Moore *et al.*, 2004; Prentice *et al.*, 2004a,b; Selak *et al.*, 2004).	A
R18	Treatment for 3 months with a GnRH agonist may be as effective as 6 months in terms of pain relief (Hornstein *et al.*, 1995). Treatment for up to 2 years with combined estrogen progestagen 'add-back' appears to be effective and safe in terms of pain relief and bone density protection (Surrey and Hornstein, 2002). However, careful consideration should be given to the use of GnRH agonists in women who may not have reached their maximum bone density.	A
R19	Depending upon the severity of disease found, ideal practice is to diagnose and remove endometriosis surgically at the same time, provided that pre-operative adequate consent has been obtained (Redwine and Wright, 2001; Abbott *et al.*, 2003; Chapron *et al.*, 2003b; Fedele *et al.*, 2004).	GPP
R20	Ablation of endometriotic lesions plus laparoscopic uterine nerve ablation (LUNA) in minimal–moderate disease reduces endometriosis-associated pain at 6 months compared to diagnostic laparoscopy; the smallest effect is seen in patients with minimal disease (Jacobson *et al.*, 2004a). However, there is no evidence that LUNA is a necessary component, as LUNA by itself has no effect on dysmenorrhoea associated with endometriosis (Vercellini *et al.*, 2003a).	A
R21	Endometriosis-associated pain can be reduced by removing the entire lesions in severe and deeply infiltrating disease. If a hysterectomy is	GPP

	performed, bilateral salpingo-oophorectomy should also be considered (Namnoum *et al*., 1995), provided that all visible endometriotic tissue is removed at the same time (Lefebvre *et al*., 2002).	
R22	Treatment with danazol or a GnRH agonist for 6 months after surgery reduces endometriosisassociated pain and delays recurrence at 12 and 24 months compared with placebo and expectant management. However, postoperative treatment with a COC is not effective (Telimaa *et al*., 1987; Parazzini *et al*., 1994; Hornstein *et al*., 1997; Bianchi *et al*., 1999; Morgante *et al*., 1999; Vercellini *et al*., 1999b; Muzii *et al*., 2000; Busacca *et al*., 2001).	A
R23	Hormone replacement therapy (HRT) is recommended after bilateral oophorectomy in young women but the ideal regimen is unclear. Adding a progestagen after hysterectomy is unnecessary but should protect against the unopposed action of estrogen on any residual disease. This theoretical benefit must be balanced against the small risk of recurrent disease (Matorras *et al*., 2002) and the increase in breast cancer risk reported to be associated with both tibolone and combined estrogen and progestagen HRT (Beral and Million Women Study Collaborators, 2003).	D
R24	Suppression of ovarian function to improve fertility in minimal–mild endometriosis is not effective and should not be offered for this indication alone (Hughes *et al*., 2004). There is no evidence of its effectiveness in more severe disease.	A
R25	Ablation of endometriotic lesions plus adhesiolysis to improve fertility in minimal–mild endometriosis is effective compared to diagnostic laparoscopy alone (Jacobson *et al*., 2004b).	A
R26	No RCT or meta-analyses are available to answer the question whether surgical excision of moderate–severe endometriosis enhances pregnancy rates. Based upon three studies (Adamson *et al*., 1993; Guzick *et al*., 1997; Osuga *et al*., 2002) there seems to be a negative correlation between the stage of endometriosis and the spontaneous cumulative pregnancy rate after surgical removal of endometriosis, but statistical significance was only reached in one study (Osuga *et al*., 2002).	B
R27	Laparoscopic cystectomy for ovarian endometriomas >4 cm diameter improves fertility compared to drainage and coagulation (Beretta *et al*., 1998; Chapron *et al*., 2002) Coagulation or laser vaporization of endometriomas without excision of the pseudocapsule is associated with a significantly increased risk of cyst recurrence (Vercellini *et al*., 2003b).	A
R28	Treatment with danazol or a GnRH agonist after surgery does not improve fertility compared with expectant management (Parazzini *et al*., 1994; Bianchi *et al*., 1999; Vercellini *et al*., 1999b; Busacca *et al*., 2001).	A
R29	Treatment with intrauterine insemination (IUI) improves fertility in minimal–mild endometriosis: IUI with ovarian stimulation is effective but the role of unstimulated IUI is uncertain (Tummon *et al*., 1997).	A
R30	IVF is appropriate treatment especially if tubal function is compromised, if there is also male factor infertility, and/or other treatments have failed.	B

R31	IVF pregnancy rates are lower in patients with endometriosis than in those with tubal infertility (Barnhart *et al*., 2002).	A
R32	Laparoscopic ovarian cystectomy is recommended if an ovarian endometrioma ≥4 cm in diameter is present to confirm the diagnosis histologically; reduce the risk of infection; improve access to follicles and possibly improve ovarian response. The woman should be counselled regarding the risks of reduced ovarian function after surgery and the loss of the ovary. The decision should be reconsidered if she has had previous ovarian surgery.	GPP
R33	Prolonged treatment with a GnRH agonist before IVF in moderate–severe endometriosis should be considered and discussed with patients because improved pregnancy rates have been reported (Rickes *et al*., 2002; Surrey *et al*., 2002).	A
R34	There is evidence from two systematic reviews suggesting that high frequency transcutaneous electrical nerve stimulation (TENS), acupuncture, vitamin B1 and magnesium may help to relieve dysmenorrhoea (Proctor and Murphy, 2004; Proctor *et al*., 2004). Whether such treatments are effective in endometriosis associated dysmenorrhoea is unknown.	D
R35	Many women with endometriosis report that nutritional and complementary therapies such as reflexology, traditional Chinese medicine, herbal treatments, homeopathy etc., do improve pain symptoms. Whilst there is no evidence from RCTs in endometriosis to support these treatments, they should not be ruled out if the woman feels that they could be beneficial to her overall pain management and/or quality of life, or work in conjunction with more traditional therapies.	GPP
R36	Patient self-help groups can provide invaluable counselling, support and advice. The website www.endometriosis.org/support.html provides a comprehensive list of all the self-help groups in the world.	GPP

Strength of evidence

Grade A:	Directly based on level 1 evidence
Grade B:	Directly based on level 2 evidence or extrapolated recommendation from level 1 evidence
Grade C:	Directly based on level 3 evidence or extrapolated recommendation from either level 1 or level 2 evidence
Grade D:	Directly based on level 4 evidence or extrapolated recommendation from either level 1, 2, or 3 evidence
Grade GPP:	Good practice point based upon the views of the Guideline Development Group

Hierarchy of evidence

**Level**	**Evidence**
1a	Systematic review and meta-analysis of randomised controlled trials
1b	At least one randomised controlled trial
2a	At least one well-designed controlled study without randomisation
2b	At least one other type of well-designed quasi-experimental study
3	Well-designed, nonexperimental, descriptive studies, such as comparative studies, correlation studies, or case studies
4	Expert committee reports or opinions and/or clinical experience of respected authorities
