# Supplementary material for: The European Society of Human Reproduction and Embryology guideline for the diagnosis and treatment of endometriosis: an electronic guideline implementability appraisal
Source: Implement Sci. 2011 Jan 19;6:7. doi: 10.1186/1748-5908-6-7 (PMC3034686; doi:10.1186/1748-5908-6-7)
Supplement: Additional file 2 — Appendix 2 - Questions of the guideline implementability appraisal (GLIA) instrument. [file 1748-5908-6-7-S2.PDF]

Appendix 2

Questions of the guideline implementability appraisal (GLIA) instrument

**Global considerations**	
Q1	Do the organization(s) and author(s) who developed the guideline have credibility with the intended users of the guideline?
Q2	Is the patient population eligible for the guideline clearly defined?
Q3	Does the guideline document suggest possible strategies for dissemination and implementation?
Q4	Is the guideline supported with tools for application e.g., a summary document, a quick reference guide, educational tools, patients' leaflets, online resources or computer software?
Q5	If any guideline recommendations are considered more important than others, does their presentation or formatting reflect this?
Q6	Is it clear in what sequence the recommendations should be applied?
Q7	Is the guideline internally consistent, i.e., without contradictions between recommendations or between text recommendations and flowcharts, summaries, patient education materials, etc.?
**Decidability**	
Q8	Would the guideline's intended audience consistently determine whether each condition in the recommendation has been satisfied? That is, is each and every condition described clearly enough so that reasonable practitioners would agree when the recommendation should be applied?
Q9	Are all reasonable combinations of conditions accounted for, i.e., is the recommendation comprehensive?
Q10	If there are more than one condition in the recommendation, is the logical relationship among all conditions (ANDs and ORs) clear?
**Executability**	
Q11	Is the recommended action (what to do) stated specifically and unambiguously? That is, would members of the intended audience execute the action in a consistent way? In situations where two or more options are offered, the executability criterion is met if the user would select an action only from the choices offered.
Q12	Is sufficient detail provided or referenced (about how to do it) to allow the intended audience to perform the recommended action, given their likely baseline knowledge and skills?
**Effect on process of care**	
Q13	Can the recommendation be carried out by current non-performers without substantial increases in provider time, staff, equipment, etc.?
Q14	Can the recommendation be tried without full provider commitment? For example, buying and installing expensive equipment to comply with a recommendation is not easily reversible.
**Presentation & formatting**	
Q15	Is the recommendation easily identifiable, e.g., summarized in a box, typed in bold, underlined, presented as an algorithm, etc.?
Q16	Is the recommendation (and its discussion) concise?

**Measurable outcomes**	
Q17	Can criteria be extracted from the guideline that will permit measurement of adherence to this recommendation? Measurement of adherence requires attention to both the actions performed and the appropriateness of the circumstances under which they are performed.
Q18	Can criteria be extracted from the guideline that will permit outcomes of this recommendation to be measured?
**Apparent validity**	
Q19	Is the justification for the recommendation stated explicitly?
Q20	Is the quality of evidence that supports the recommendation explicitly stated?
**Novelty / innovation**	
Q21	Can the recommendation be performed by the guideline's intended users without the acquisition of new competence (knowledge, skills)?
Q22	Is the recommendation compatible with existing attitudes and beliefs of the guideline's intended users?
Q23	Is the recommendation consistent with patient expectations? In general, patients expect their concerns to be taken seriously, benefits of interventions to exceed risks, and adverse outcomes to fall within an acceptable range.
**Flexibility**	
Q24	Does the recommendation specify patient or practice characteristics (clinical and non-clinical) that require (or permit) individualization? For example, immediate angioplasty and MR imaging may not be available in all settings.
Q25	Does the recommendation consider coincident drug therapy and common co morbid conditions?
Q26	Is there an explicit statement by the guideline developer regarding the strength of this recommendation? Note: There is a difference between quality of evidence (item 20) and stringency of a policy. Potential statements to satisfy this criterion might include "Strong recommendation", "Standard", Clinical option", etc.
Q27	If patient preference is considered does the recommendation propose mechanisms for how it is to be incorporated?
**Computability**	
Q28	Are all patient data needed for this recommendation available electronically in the system in which it is to be implemented?
Q29	Is each condition of the recommendation defined at a level of specificity suitable for electronic implementation?
Q30	Is each recommended action defined at a level of specificity suitable for electronic implementation?
Q31	Is it clear by what means a recommended action can be executed in an electronic setting, e.g., creating a prescription, medical order, or referral, creating an electronic mail notification, or displaying a dialog box?
