# Supplementary material for: The European Society of Human Reproduction and Embryology guideline for the diagnosis and treatment of endometriosis: an electronic guideline implementability appraisal
Source: Implement Sci. 2011 Jan 19;6:7. doi: 10.1186/1748-5908-6-7 (PMC3034686; doi:10.1186/1748-5908-6-7)
Supplement: Additional file 3 — Appendix 3 - Example of scoring. [file 1748-5908-6-7-S3.PDF]

Appendix 3 Table of the 31 questions of the GLIA instrument with a score example

	Global Score				Score Rec. 1				Score Rec. 2			
	Y	N	NA	?	Y	N	NA	?	Y	N	NA	?
**Global consideration**												
1. Do guideline developers have credibility with the users?	X											
2. Is patient population clearly defined?	X											
3. Are there possible strategies for implementation?		X										
4. Is the guideline supported with tools for application?		X										
5. Does presentation of recommendations reflect importance?		X										
6. Is the sequence of applying the recommendations clear?	X											
7. Is the guideline internally consistent?				X								
**Decidability**												
8. Is every condition described clearly enough?					X						X	
9. Are all reasonable combinations of conditions accounted for?					X						X	
10. Is the logical relationship among all conditions clear?					X						X	
**Executability**												
11. Is specifically and unambiguously stated what to do?						X			X			
12. Is sufficient detail provided, how to do the stated action?					X				X			
**Effect on process of care**												
13. Can the recommendation be carried out by non-performers?								X		X		
14. Can the recommendation be tried without full provider commitment?								X				X
**Presentation & formatting**												
15. Is the recommendation easily identifiable?					X				X			
16. Is the recommendation (and its discussion) concise?					X					X		
**Measurable outcomes**												
17. Can criteria be extracted to measure adherence?					X				X			
18. Can criteria be extracted to measure outcomes?					X						X	
**Apparent validity**												
19. Is the justification for the recommendation stated explicitly?					X				X			
20. Is the quality of evidence stated explicitly?					X				X			
**Novelty/Innovation**												
21. Can the recommendation be performed without the acquisition of new competence?					X					X		
22. Is the recommendation compatible with beliefs of the users?					X					X		
23. Is the recommendation consistent with patient expectations?					X							X

	Global Score				Score Rec. 1				Score Rec. 2			
	Y	N	NA	?	Y	N	NA	?	Y	N	NA	?
**Flexibility**												
24. Are patient or practice characteristics specified?					X					X		
25. Are coincident drug therapy & co morbid conditions considered?					X					X		
26. Is there a statement of the developer regarding the strength of the recommendation?						X				X		
27. Are there proposed mechanisms to incorporate patient preference?							X				X	
**Computability**												
28. Are all patient data available electronically?					X						X	
29. Is each condition defined for electronic implementation?					X						X	
30. Is each action defined for electronic implantation?					X						X	
31. Is described how actions can be executed in an electronic setting?					X						X	

(grey) No scoring because these questions are applicable for the guideline as a whole
