# Supplementary material for: The European Society of Human Reproduction and Embryology guideline for the diagnosis and treatment of endometriosis: an electronic guideline implementability appraisal
Source: Implement Sci. 2011 Jan 19;6:7. doi: 10.1186/1748-5908-6-7 (PMC3034686; doi:10.1186/1748-5908-6-7)
Supplement: Additional file 4 — Appendix 4 - Final report. [file 1748-5908-6-7-S4.PDF]

Appendix 4 Final report

Barrier | Facilitator | Borderline barrier | N=No; Y=Yes; X=Not applicable | Not applicable

Recommendations | Global question | Nonrecommendation | Excluded item

eGLIA question		1	2	3	4	5	6	7	8	9	10	11	12	13	14	15	16	17	18	19	20	21	22	23	24	25	26	27	28	29	30	31	32	33	34	35	36
Q1																																					
Q2																																					
Q3																																					
Q4																																					
Q5																																					
Q6																																					
Q7																																					
Q8																																					
Q9																																					
Q10													X																								
Q11														N										Y												N	
Q12																																				N	
Q13										Y		N																									
Q14			Y																																		
Q15																																					
Q16																																					
Q17																								Y												N	
Q18								Y																													
Q19																																					
Q20																																					
Q21																																					
Q22																																					
Q23																																					
Q24																																					
Q25												X																									
Q26																																					
Q27																																					
Q28																																					
Q29																																					
Q30																																					
Q31																																					
